# Supplementary material for: Disialoganglioside GD2-Targeted Near-Infrared Photoimmunotherapy (NIR-PIT) in Tumors of Neuroectodermal Origin
Source: Pharmaceutics. 2022 Sep 24;14(10):2037. doi: 10.3390/pharmaceutics14102037 (PMC9612122; doi:10.3390/pharmaceutics14102037)
Supplement: Supplementary file 1 [file pharmaceutics-14-02037-s001.zip › pharmaceutics-1900800-supplementary.pdf]

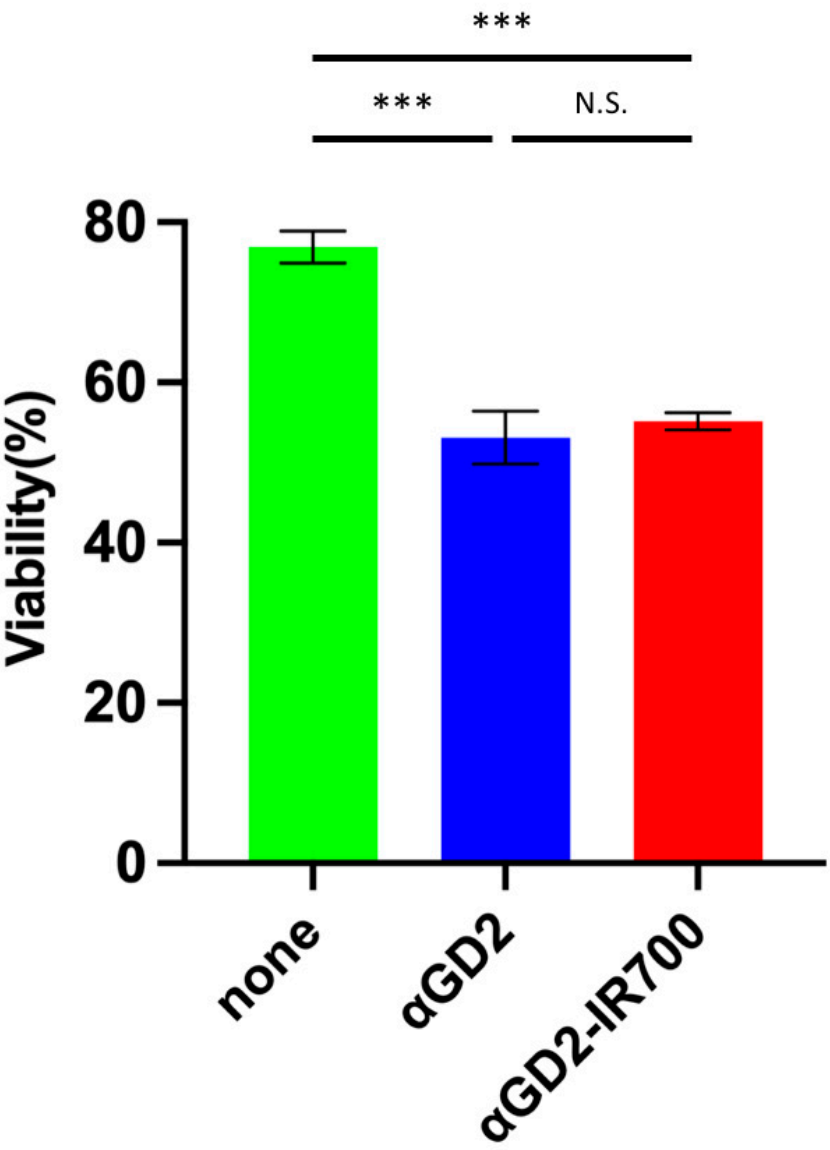

Figure S1. Cell viability after incubation with αGD2 antibody. Cell viability after incubation with no antibody, αGD2 antibody, or αGD2-IR700. Cell viability was measured by PI staining. Data are shown as mean ± SEM (n = 4, *** $p < 0.001$; N.S., not significant).
